# Cortical Response Similarities Predict which Audiovisual Clips Individuals Viewed, but Are Unrelated to Clip Preference

**DOI:** 10.1371/journal.pone.0128833

**Published:** 2015-06-01

**Authors:** David A. Bridwell, Cullen Roth, Cota Navin Gupta, Vince D. Calhoun

**Affiliations:** 1 The Mind Research Network, Albuquerque, New Mexico, United States of America; 2 Department of Mathematics and Statistics, University of New Mexico, Albuquerque, New Mexico, United States of America; 3 Department of Biology, IMSD, University of New Mexico, Albuquerque, New Mexico, United States of America; 4 Program in Genetics and Genomics, Duke University, North Carolina, United States of America; 5 Department of ECE, University of New Mexico, Albuquerque, New Mexico, United States of America; Harvard Medical School/Massachusetts General Hospital, UNITED STATES

## Abstract

Cortical responses to complex natural stimuli can be isolated by examining the relationship between neural measures obtained while multiple individuals view the same stimuli. These inter-subject correlation’s (ISC’s) emerge from similarities in individual’s cortical response to the shared audiovisual inputs, which may be related to their emergent cognitive and perceptual experience. Within the present study, our goal is to examine the utility of using ISC’s for predicting which audiovisual clips individuals viewed, and to examine the relationship between neural responses to natural stimuli and subjective reports. The ability to predict which clips individuals viewed depends on the relationship of the EEG response across subjects and the nature in which this information is aggregated. We conceived of three approaches for aggregating responses, i.e. three assignment algorithms, which we evaluated in Experiment 1A. The aggregate correlations algorithm generated the highest assignment accuracy (70.83% chance = 33.33%) and was selected as the assignment algorithm for the larger sample of individuals and clips within Experiment 1B. The overall assignment accuracy was 33.46% within Experiment 1B (chance = 06.25%), with accuracies ranging from 52.9% (*Silver Linings Playbook*) to 11.75% (*Seinfeld*) within individual clips. ISC’s were significantly greater than zero for 15 out of 16 clips, and fluctuations within the delta frequency band (i.e. 0-4 Hz) primarily contributed to response similarities across subjects. Interestingly, there was insufficient evidence to indicate that individuals with greater similarities in clip preference demonstrate greater similarities in cortical responses, suggesting a lack of association between ISC and clip preference. Overall these results demonstrate the utility of using ISC’s for prediction, and further characterize the relationship between ISC magnitudes and subjective reports.

## Introduction

Individuals experience a broad range of perceptual and cognitive events throughout the day. This complexity is approximated by the complexity of the experience of watching short movies or audiovisual clips, which may invoke a greater array of emotional and perceptual experiences than traditional cognitive experiments [1]. However, the diversity of cognitive experience is paralleled by the resultant diversity in neural responses to the stimulus [2,3]. In order to better characterize these responses, it is important to understand the relationship between neural responses to natural stimuli and subjective reports, and to develop approaches to understand the information that may be retrieved (i.e. decoded) from cortical responses to complex stimuli.

Cortical responses may emerge from natural stimuli as either evoked oscillations (i.e. evoked potentials) from the stimuli, or phase entrainment (i.e. phase tracking) to the stimulus features [4,5]. These responses may be mediated in part by individual’s preference toward the stimulus, which may be interrelated with their attentional and emotional engagement with the stimulus content [6]. These factors contribute to the characteristics of electroencephalography (EEG) or magnetoencephalography (MEG) responses that may be preserved across subjects viewing the same stimulus, which would facilitate the ability to integrate information across multiple subjects for predicting the audiovisual clip that an individual viewed.

The relationship between neural time series may be examined across individuals using Pearson’s correlation coefficient, or inter-subject correlation’s (ISC’s) [7–9]. Within the present study, we examine the utility of using EEG ISC’s for predicting which audiovisual clips individuals viewed. The approach emphasizes the relationship between responses measured across multiple individuals viewing the same stimulus, providing a means to extract information from complex stimuli without explicitly extracting the low and high level auditory and visual features presented. In this manner, the utility of predicting which clips individuals viewed depends on the relationship of the EEG response across subjects and the nature in which this information is aggregated. We conceived of three approaches for aggregating responses, i.e. three assignment algorithms, which we implement to examine the utility of ISC’s for assigning individual EEG segments to one of the 16 audiovisual clips presented.

Since ISC’s may emerge from similarities in the direct neural response to the audiovisual inputs, as well as similarities in their emergent perceptual experience [10,11], we additionally examined whether individuals with similar clip preferences had similar cortical responses to the clips. These findings add to previous studies which focus on the relationship between ISC’s and individuals subjective experience of natural stimuli, including whether they perceive the clip to invoke negative or positive emotions (i.e. the clips valence), whether they perceive the clip to invoke a low or high level of arousal or emotional response, and whether they enjoyed the clip content [6,12,13].

The relationship between ISC’s and valence and arousal appears mixed, however, with relationships demonstrated in some cases [6], but not others [13]. These differences may be related to the differences in clip content between the two studies, with Nummenmaa et al., 2012 presenting 13 short (i.e. ~30 sec to ~2 min.) clips characterized by discrete changes in valence and arousal, while Jääskeläinen et al., 2008 presented a continuous 30 minute clip with fewer discrete changes in valence/arousal. Thus, ISC’s may show a greater increase when the eliciting events are well defined, but may be insensitive to subtle fluctuations in valence/arousal from longer segments primarily characterized by low arousal or neutral valence.

The nuanced relationship between subjective reports and ISC’s is further demonstrated by a recent study by Dmochowski et al., 2014. The authors examined the relationship between EEG ISC magnitudes (measured while individuals watched Super Bowl advertisements) with the preference of the broad population and the preference of the individuals within the study. They found that the EEG response similarities explained 66% of the variance of population ratings, but only 26% of the variance of the individuals own preferences. Thus, EEG ISC’s appear better at predicting the preferences expressed by a broad audience than the preferences obtained directly from the individuals in which the ISC’s are measured [12].

Within the present study, our primary goal is to examine the utility of using ISC’s for predicting which clips individuals viewed. Within Experiment 1A, we examine the prediction accuracy of three different assignment algorithms which emphasize different aspects of similarity across subjects. The algorithm is implemented within Experiment 1B in a larger sample viewing a diverse array of audiovisual clips. Within Experiment 1B, we recommend the best performing algorithm for future studies implementing EEG ISC’s for prediction. In order to understand the relationship between ISC’s and individual’s perceptual experience, within Experiment 1B we additionally examined whether ISC’s were related to individual preference. We were unable to demonstrate a statistical relationship between ISC’s and preference, which suggests that this measure of subjective experience is unrelated to cortical response similarities across subjects.

## Materials and Methods (Experiment 1A)

### Participants

Two subjects (2 males, 22 and 31 years old) were recruited to participate in *algorithm selection* sessions in order to select the assignment algorithm to predict which video clips subjects watched within the main experiment. Each individual had normal or corrected to normal vision and had no family history of mental illness. Both participants were enrolled in protocols approved by the University of New Mexico Institutional Review Board (HRRC/MCIRB), and provided informed written consent prior to the first session at the Mind Research Network.

### Design and Stimuli

The video clips were presented on a computer using VLC media player. Participants were seated 56 cm from the computer screen, and the VLC media player window comprised 13.2 degree visual angle (dva) horizontally and 7.4 dva vertically. The audio was presented to the individual through speakers placed adjacent the monitor. On average, the audio intensity was ~ 90 decibels (db) across all clips, and the maximum intensity never exceeded ~95 db (calibrated 4 inches away from the speakers). Two individuals participated in four sessions each consisting of three ~4:56 min video clips (Table 1) presented in a fixed order. The 3 clips for algorithm selection were primarily composed of comedic speech. Many of the 16 test clips contain comedic content, and the auditory information within the test clips is predominantly speech, thus there is considerable overlap between the content of the 3 clips for algorithm selection (Experiment 1A) and the content of the 16 test clips (Experiment 1B).

**Table 1:** Filmography: Detailed information about the video clips displayed within the algorithm selection sessions.

Clip	Title	Year	Directed/Created by	Clip Quote	Brief Description
1	Bill Cosby, Himself	1983	Bill Cosby	“My wife and I were intellectuals, before we had children”	Bill Cosby describes natural childbirth
2	Eddie Izzard Glorious	1997	Peter Richardson, Eddie Izzard	“The glove compartment is a lie in cars. You never open it and go, pairs of gloves!”	Eddie Izzard talks about lawn mowers, toasters, and showers.
3	Zach Galifianakis: Live at the Purple Onion	2006	Michael Blieden, Zach Galifianakis	“I was named after my granddad. Yes, my full name is Zach Granddad Galifianakis..”	Zach Galifianakis entertains the audience with one-liners.

### EEG acquisition and preprocessing

EEG responses were collected with two 4 channel CamNtech Actiwave mobile EEG devices (sampling rate: 128 Hz). The eight electrodes where placed on the participant’s scalp using the standard 10–20 locations F3, Fz, F4, Cz, P3, P4, O1, and O2, with a left mastoid reference (e.g. see [14]). EEG analysis was conducted in MATLAB using custom and built-in functions. The EEG data was linearly detrended and forward and backward filtered with a Butterworth filter (bandpass: 0.01 to 50 Hz).

The continuous EEG recordings were segmented into individual segments for each ~4:56 min video clip. The time-courses were visually inspected for each electrode and segment (i.e. clip) and artifactual time-courses were marked for removal before averaging across time series, as described in detail below. Artifactual segments typically contained multiple segments of large amplitude fluctuations. 4.87 out of 24 (3 clips × 8 electrodes) segments were removed on average.

### Correlation and video clip assignments

Our goal was to assign a particular EEG segment measured with a given individual to 1 of the 3 video clips. To illustrate this specifically, consider determining which video clip an individual watched while we recorded 1 of the 3 [8 channels × 37940 samples] EEG segments in an individual session. The general approach was to assign this segment, which we refer to as the withheld segment, to one of the three clips using the segments recorded in the other sessions (i.e. sessions 2–8), when subjects watched each of the three clips. In this example, we refer to the segments from sessions 2–8 as the reference segments.

**Fig 1:**
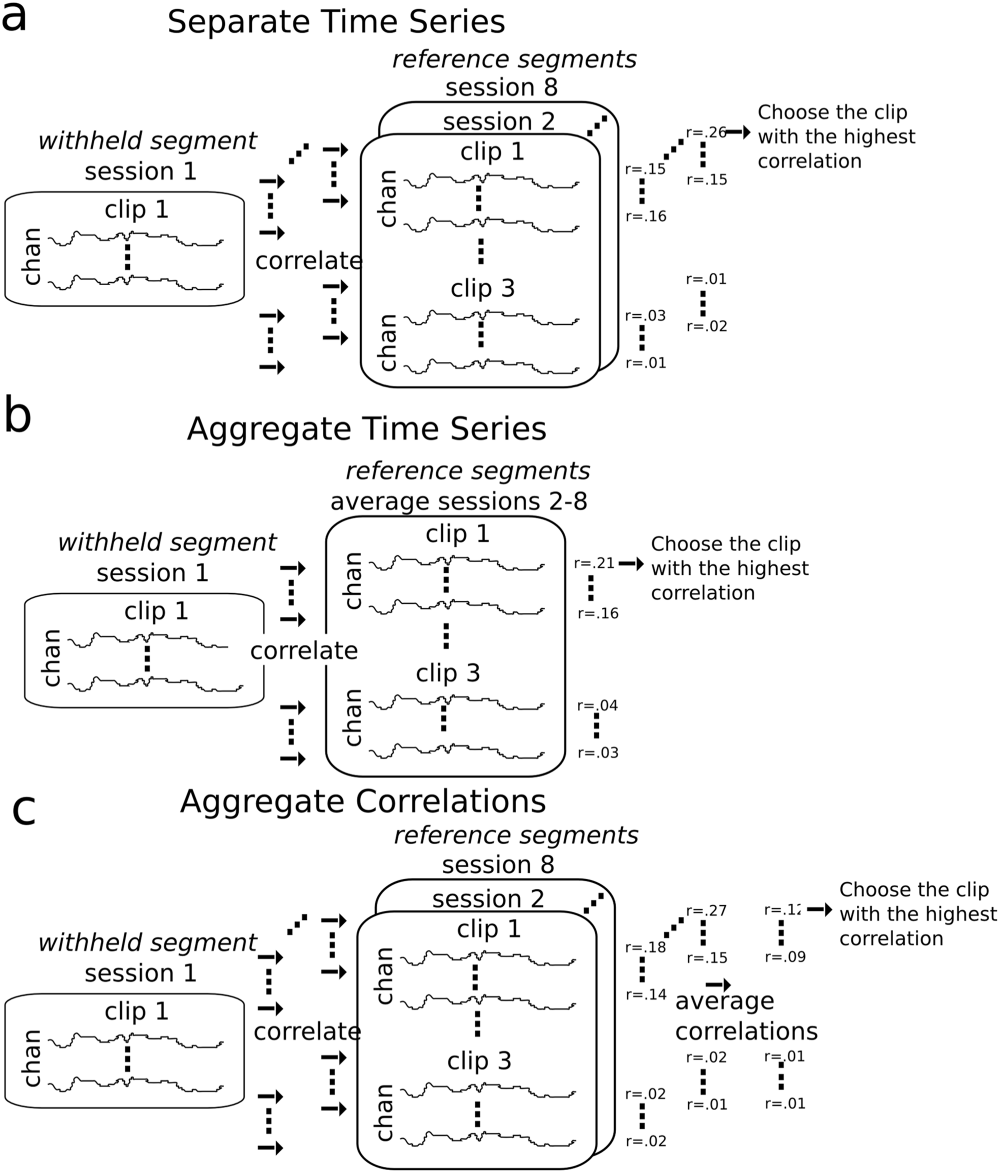
Three assignment algorithms. Individual [channel × time] segments were assigned to one of the video clips using the separate time series approach (a), the aggregate time series approach (b) or the aggregate correlations approach (c). Within (a), electrode-wise correlations are computed between the *withheld segment* and the *reference segments* (i.e. the segments recorded within all other sessions and clips). The reference segment which contributes to the highest correlation was identified, and the clip that corresponds to that segment was assigned to the withheld segment. Within b, the reference segments are averaged electrode-wise across identical clips prior to computing correlations, and within c, the electrode-wise correlations are averaged across sessions that correspond to the same clip. The clips are assigned in the same manner as a. The process is repeated using each segment as the withheld segment, generating an assignment for every segment. These approaches emphasize different aspects of similarities across subjects, where (a) is advantageous in instances where only a subset of subjects demonstrate a similar response on a given clip, (b) is advantageous in instances where subjects demonstrate similar cortical responses within a clip, and when these similarities would be obscured when averaging across time series (as in c). C preserves the similarities in the time series across individuals, and is thus advantageous when the majority of individual’s demonstrate similar responses to the clips, and averaging time series enhances these similarities (i.e. the signal).

The relationship between the withheld segment and the reference segments were examined by calculating the electrode-wise Pearson correlation coefficient between the time series. We conceived of three potential ways in which the time series may be related across subjects, which motivated the three assignment algorithms that were evaluated within Experiment 1A (Fig 1). The algorithms differ with respect to whether the reference segments were averaged before calculating the correlation with the withheld segment, and whether the correlation between the reference and withheld segments were averaged prior to the assignment. These three approaches, which we term the aggregate time series approach, the separate time series approach, and the aggregate correlations approach, are described in detail below.

In the separate time series approach (Fig 1a) electrode-wise correlations were computed between the withheld segment and the time series of the individual reference segments generating 168 correlation values (3 clips × 8 electrodes × 7 remaining sessions). The maximum correlation was identified and the video clip that corresponded to that EEG segment was assigned to that particular withheld segment. This approach preserves the relationship between pairs of subjects, which is advantageous in instances where only a subset of subjects demonstrate a similar response to a given clip.

Within the aggregate time series approach (Fig 1b) electrode-wise correlations were computed between the withheld segment and the time series of the reference segments from the other sessions after averaging the reference segments across sessions that correspond to the same video clip. These correlations were calculated for each of the three clips and each of the 8 electrodes generating 24 correlation values. The maximum correlation was identified and the video clip that corresponded to the EEG segment that contributed to the calculation was assigned to that particular withheld segment. This approach is advantageous when the majority of subjects have similar responses to the audiovisual content. Averaging across subjects enhances these similarities (i.e. the signal), and attenuates the noise, as in conventional event related potential (ERP) studies.

In the aggregate correlations approach (Fig 1c) correlations were computed between the withheld segment and the time series of the individual reference segments. These correlation values were then averaged across the reference segments that correspond to the same clip, generating 24 correlation values for evaluation (3 clips × 8 electrodes). As in the other approaches, the maximum correlation was identified and the video clip that corresponded to the EEG segment that contributed to the calculation was assigned to that particular withheld segment. This approach integrates information from multiple subjects similar to the aggregate time series approach, but preserves similarities in the evoked potential within subject pairs which might be obscured when averaging across all subjects.

Within each of the three approaches, assignments were computed for each of the 24 withheld segments, and assignment accuracy was evaluated by determining the percentage of segments which were correctly assigned. The algorithm with the highest assignment accuracy was then used to predict which of the 16 video clips corresponded to each segment within the main experiment.

## Results (Experiment 1A)

### Algorithm selection and assignment accuracy

Fourteen, 13, and 17 of the EEG segments were correctly assigned to 1 of 3 clips using the separate time series (Fig 1a), aggregate time series (Fig 1b), and aggregate correlations (Fig 1c) assignment algorithms respectively. Among the 24 recorded segments, assignment accuracies were 58.33%, 54.17%, and 70.83% (chance = 33.33%), with the highest assignment accuracy with the aggregate correlations approach. Thus, we observed a 30.76% and 21.43% increase in assignment accuracy between the aggregate correlations approach and the separate time series and aggregate time series approaches, respectively. Based on these findings, the aggregate correlations approach was selected as the assignment algorithm within the data collected in Experiment 1B.

## Materials and Methods (Experiment 1B)

### Participants

Eighteen subjects (12 males) between the ages 20 and 35 were recruited to participate. One individual was excluded due to artifactual EEG, bringing the total number of participants to 17. Each individual had normal or corrected to normal vision and had no family history of mental illness. All participants were enrolled in protocols approved by the University of New Mexico Institutional Review Board (HRRC/MCIRB), and provided informed written consent prior to the first session at the Mind Research Network.

### Design and Stimuli

The viewing distance and audio levels were identical to Experiment 1A. Eighteen individuals viewed sixteen clips (Table 2) within a single session, with clip durations between 3:55 and 3:59 min. The 16 clips were displayed in blocks of four, and subjects took a short break between each block. The order of the 16 clips were balanced across subjects using a Latin square design. The Latin square consisted of a 16 × 16 matrix with the elements 1, 2, … 16 such that no element repeated within a given row or column. Subjects were instructed to silence their cell phones, to minimize movements and to “relax and enjoy the show”. Subjects ranked the 16 clips at the end of the session by preference, rated each clip on a scale of 1 to 5, with 1 representing “hated it” and 5 representing “loved it”, and indicated whether they had seen the content of each clip before.

**Table 2:** Filmography: Detailed information about the video clips displayed within the main experiment.

Clip	Title	Year	Directed/Created by	Content Summary	Clip Quote	Brief Description
1	50/50	2011	Jonathan Levine	Romance	“Did I just score your digits”	A client expresses interest in his therapist.
2	Requiem for a Dream	2000	Darren Aronofsky	Drama	“Are you on uppers ma?	A son confronts his mother about taking pills.
3	Who’s Afraid of Virginia Woolf	1966	Mike Nichols	Drama	“Decency Martha”	A couple argues in front of their guests.
4	The Office(Season 2, Ep. 1)	2005	Ricky Gervais, Stephen Merchant, Greg Daniels	Romance	“And the award for whitest sneakers goes to:”	Two co-workers express their latent romantic attractions.
5	Vanilla Sky	2001	Cameron Crowe	Romance	“Do you ever accept any of your 12,000 proposals?”	A man flirts with a woman he met at a party.
6	Silver Linings Playbook	2012	David O. Russell	Awkward social interaction	“you say more inappropriate things than appropriate things”	A man meets and interacts awkwardly with another woman.
7	Jim Gaffigan, King Baby	2009	Troy Miller	Comedy (stand-up)	“my parents never took me camping, because they loved me”	Jim Gaffigan describes the downsides of camping
8	West Wing (Season 2, Ep. 3)	2000	Aaron Sorkin	Drama	“Nobody sits”	The president confronts a conservative radio host about gay marriage.
9	Meet Joe Black	1998	Martin Brest	Romance	“you could be her”	A man flirts with a woman sitting next to him.
10	Good Will Hunting	1997	Gus Van Sant	Social interaction	“my boy is wicked smart”	The protagonist out intellectualizes an intellectual in front of women at a bar.
11	Breaking Bad (Season 5, Ep. 6)	2012	Vince Gilligan	Awkward social interaction	“Did you also tell him about my affair?”	An awkward conversation over dinner.
12	Bill Cosby, Himself	1983	Bill Cosby	Comedy (stand-up)	“Dad is great! Gave us the chocolate cake!”	Bill Cosby describes when he fed his children chocolate cake for breakfast.
13	Friends(Season 4, Ep. 1)	1997	Marta Kauffman, David Crane	Comedy	“It stung real bad”	The friends disclose that one was stung by a jellyfish and the other peed on the sting.
14	Ellen, One Night Stand	2003	Joel Gallen	Comedy (stand-up)	“you ever walk into a plate glass window?”	Ellen describes awkward social interactions.
15	Fargo	1996	Joel Coen, Ethan Coen	Awkward social interaction	“I like you so much Marge”	A woman has an awkward conversation with an old friend.
16	Seinfeld	1998	Marty Callner	Comedy (stand-up)	“If I’m the best man why is she marrying him”	Seinfeld talks about candy, chopsticks, McDonalds, and being a best man at a wedding.

### EEG acquisition, preprocessing, and wavelet decomposition

The continuous EEG recordings were acquired (see Experiment 1A) and segmented into ~ 4:00 min intervals for each video clip. Segments were preprocessed as described in Experiment 1A (section 2.1.3). In order to determine the frequency that contributes to ISC’s, the EEG time series were decomposed into time-frequency representations using the discrete wavelet transform (DWT) prior to ISC calculations. We chose multiresolution analysis (MRA) [15,16] by DWT since it provides an improved localization and resolution of EEG features compared to conventional digital filtering [17]. The biorthogonal spline mother wavelet was used since they have optimal mathematical characteristics (i.e. they are anti-symmetric, smooth, and have optimal time-frequency resolution (compact support)) [18,19] and since their shape is similar to evoked potentials [20]. Detailed descriptions of the WT are provided in [21–23]. In general, the DWT filters the input signal by multiple matched filters that are shifted and scaled. Wavelet coefficients are computed for each filter by an integral transform and the original signal is reconstructed using the inverse WT. Conceptually, patterns within the original signal are preserved when they match the pattern of the filter. Different frequencies are emphasized by scaling and shifting the filter at each level of the DWT, generating filtered representations of the original signal within the characteristic EEG frequency bands [24–26].

Five levels of DWT were implemented using the wavedec and wrcoef functions in matlab with a biorthogonal spline wavelet (bior3.9; dyadic decomposition). Each level of decomposition returns two time-frequency representations with half the frequency band of the input, a course representation (i.e. the approximation A), and a high frequency representation (i.e. the detail, D). With a 128 Hz signal, the first level of decomposition returns an approximation A containing ~0–32 Hz and its associated detail, D encompassing ~32–64 Hz. At the second level, the approximation AA is ~0–16 Hz and the detail AD is ~16–32 Hz. Following this convention, we focus analysis on the following details and approximations which approximately overlap with the characteristic EEG frequencies: delta (AAAA; ~0–4 Hz), theta (AAAD; ~4–8 Hz), alpha (AAD; ~8–16 Hz), beta (AD; ~16–32 Hz), gamma (DA: ~32–48).

### Correlation and video clip assignments

Individual [8 channels × 30721 samples] segments were assigned to 1 of the 16 video clips using the aggregate correlations algorithm (Fig 1c), i.e. the algorithm with the highest assignment accuracy within Experiment 1A.

### Dependence on the number of subjects

The assignment accuracy likely depends on the number of subjects that contribute to the reference segments in the assignment. We evaluated the nature of this dependence when N = 1, 2, 3 … 16 subjects were used as reference. When N < 16, an individual withheld segment is assigned to a particular video clip using all of the unique combinations of subjects from the pool of 16. For example, in the instance where 2 subjects are used as reference, there are 120 unique combinations of the 16 subjects available. The average and standard deviation of these assignment accuracies were examined for each unique combination of subjects, generated with N = 1, 2, 3, … 16 subjects in the assignment.

### Statistical Analysis

Statistical tests were conducted to determine whether ISC’s were significantly greater than zero for each of the 16 clips (16 permutation tests). Additional tests (Pearson’s r) were conducted to determine whether there was a relationship between ISC magnitudes and individual clip preferences, and ISC magnitudes and assignment accuracy. Statistical tests are reported as significant if p < 0.0028, which conservatively corrects for the 18 tests (i.e. with alpha = .05 and 18 tests, Bonferroni corrected p-values are = 0.0028).

## Results (Experiment 1B)

### ISC’s within different wavelet frequencies

**Fig 2:**
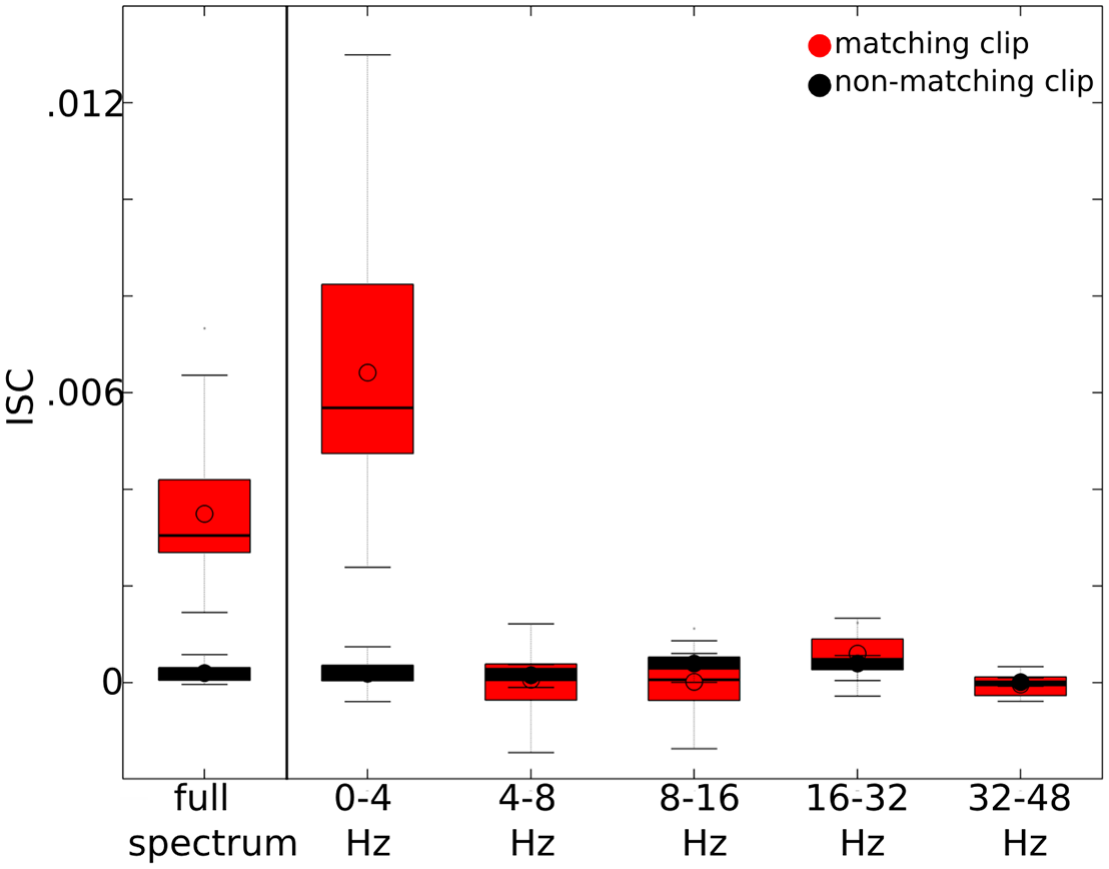
Average ISC’s within different frequencies. The average ISC was calculated for each individual across matching clips (in red) and non-matching clips (in black) across all unique subject pairs and electrodes. The distribution of individual ISC’s are indicated when computed from the full spectrum (i.e. 0–50 Hz), and when computed from signals wavelet filtered within the ~0–4 Hz, 4–8 Hz, 8–16 Hz, 16–32 Hz, and 32–48 Hz frequency bands. The upper and lower bars indicate the maximum and minimum data points, respectively. The red bar spans the first (lower) and third (upper) quartiles. The solid line indicates the median and the circle indicates the mean.

The EEG signals were filtered with DWT in order to determine which frequencies contributed to ISC’s. For a given wavelet frequency and electrode, ISC’s were computed for each pair of segments that correspond to matching clips and non-matching clips. For example, for a given pair of subjects, 16 correlations were computed among matching segment pairs (16 movies) and 120 correlations were computed among unique non-matching segment pairs. The 2048 matching correlations (16 clips × 8 electrodes × 16 remaining subjects) were averaged for each individual, and the distribution of individual averages is indicated in Fig 2 when ISC’s were computed from the broadband EEG signal (0–50 Hz) or the wavelet filtered signals.

The overall average ISC increased from .0035 with the broadband EEG signal (0–50 Hz) to .0064 with the signal filtered between 0–4 Hz (Fig 2). The other frequency bands (i.e. 4–8 Hz, 8–16 Hz, 16–32 Hz, and 32–48 Hz) have negligible contribution to ISC’s. Thus, it appears that the low frequency (i.e. delta) EEG content primarily contributes to the observed similarities in responses across subjects. Based on this finding, we focus our subsequent analysis on ISC’s computed from the 0–4 Hz filtered signal.

### Average ISC’s for each of the 16 clips

**Fig 3:**
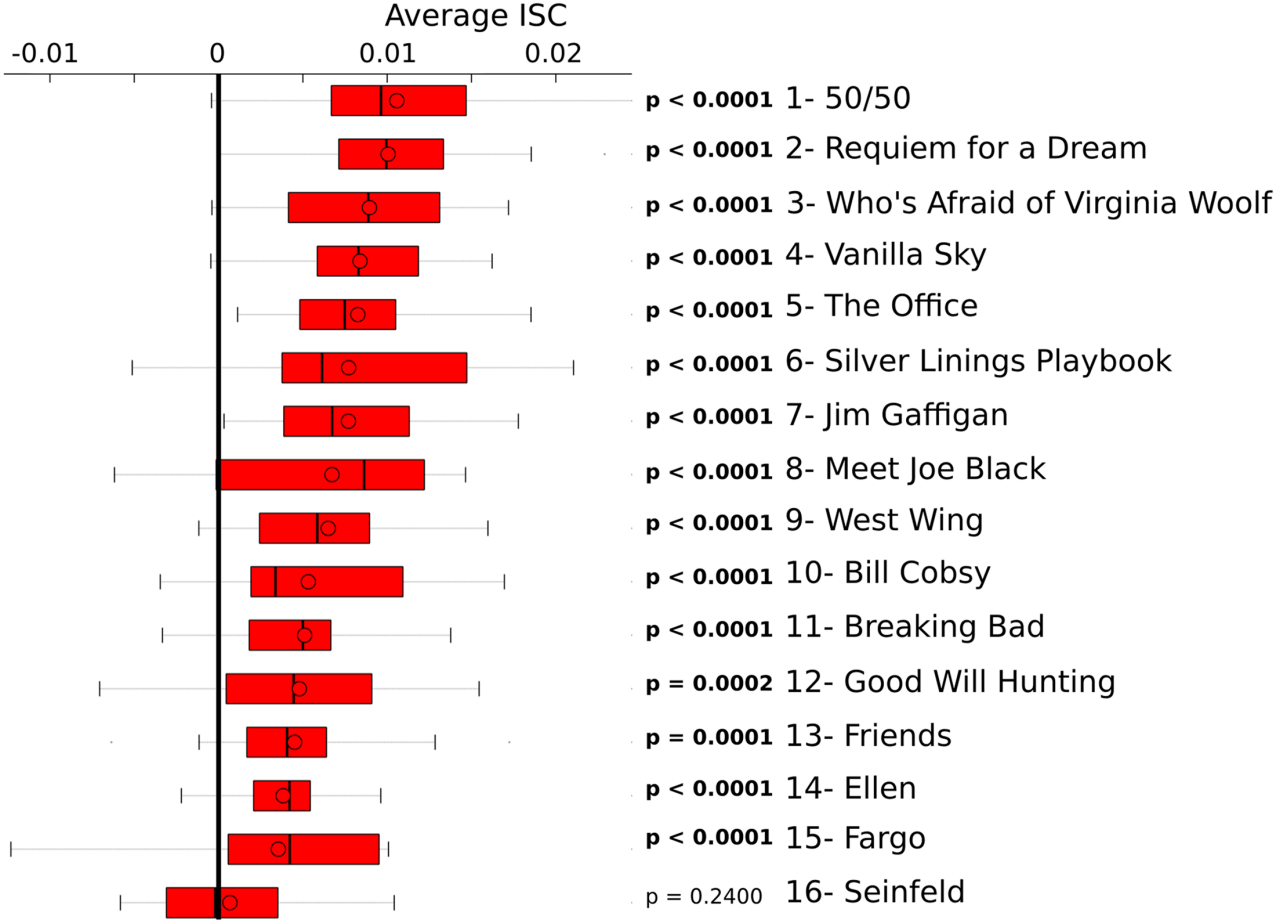
The average ISC for each video clip. ISC’s were calculated across all unique subject pairs and electrodes. The ISC's were averaged across all electrodes and subject pairs for each individual. The distribution of individual average ISC’s are indicated in each boxplot, separately for each clip. The boxplots are organized with the highest average ISC’s on top and the lowest ISC’s on bottom. ISC’s were greater than zero (permutation test: p < 0.0028) for 15 out of 16 clips. Within each boxplot, the upper and lower bars indicate the maximum and minimum data points, respectively. The red bar spans the first (lower) and third (upper) quartiles. The solid line indicates the median and the circle indicates the mean.

Individual ISC’s (N = 17) were computed for each clip by averaging the correlations computed among 128 segment pairs (8 electrodes × 16 remaining subjects). The distribution of individual average ISC’s is indicated in Fig 3. We examined whether the overall average ISC (i.e. averaged across subjects) was greater than zero by conducting a non-parametric permutation test [27,28]. The permutation distribution of ISC’s were computed in the same manner as described above, except that a given segment was correlated to a segment corresponding to different clip (i.e. a non-matched clip). The non-matching segment was drawn at random for each iteration (100,000 iterations). The non-matched permutation ICS’s were averaged across all subjects for each permutation and p-values were derived by determining the percentage of permutation ISC’s which exceed the average ISC value calculated when the segments correspond to the same clip. The average ISC was significantly above zero (p < 0.0028) for 15 out of the 16 clips. There was insufficient evidence that ISC’s were above zero when individuals watched the clip from Seinfeld (permutation test: p = 0.240).

The spatial distribution of ISC’s were examined by computing the average ICS (across all subject pairs and clips) separately for each electrode. The highest ISC’s were observed at electrode Cz (r = 0.0083) and electrode 02 (r = 0.0081). The ISC’s were 0.0055, 0.0054, 0.0073, 0.0060, 0.0065, and 0.0044 within electrodes Fz, O1, F3, F4, P3, and P4, respectively (S2 Fig). Thus, the highest ICS’s were observed over the mid central electrode and the electrode located over occipital regions.

### ISC’s and clip preference

**Fig 4:**
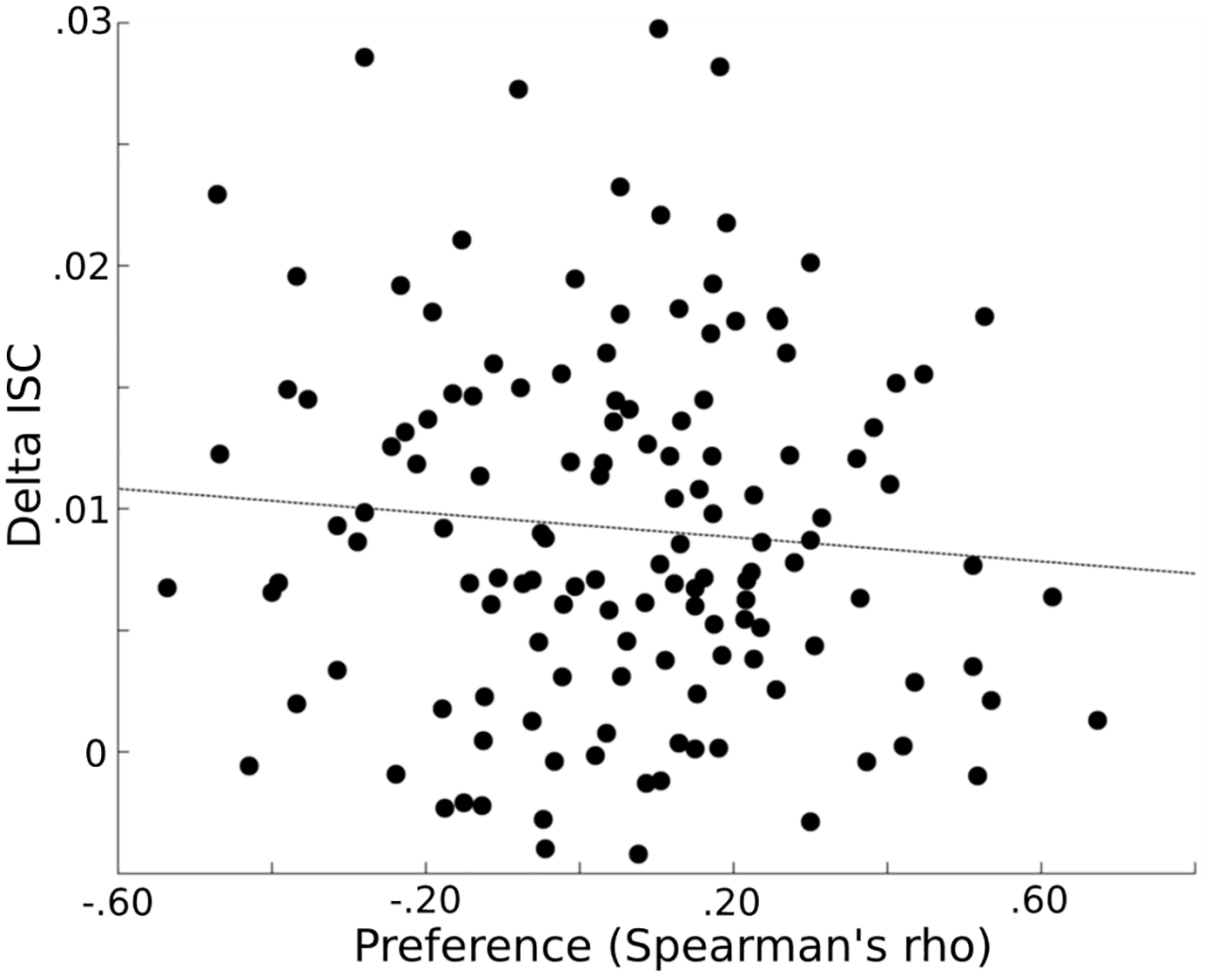
Preference and delta ISC’s. Similarities in preference (x-axis) (the Spearman’s rho between clip rankings for each subject pair) are plotted against similarities in delta EEG responses (y-axis) (represented by the average ISC). There was insufficient evidence of a relationship among clip preference and ISC’s (r(135) = -0.08; p = 0.34).

The similarity in preferences between each subject pair was calculated by computing the Spearman rank correlation coefficient between their rankings for the 16 video clips. If ISC’s are related to individual preferences, then subjects with similar preferences, as reflected by larger Spearman’s rho values, would demonstrate a greater similarity in their EEG response to the clips, as reflected in larger ISC’s. The Spearman’s rho and average delta-band ISC’s (i.e. across the 16 movies and 8 electrode) values were uncorrelated across the subject pairs (r(135) = -0.08; p = 0.34), indicating insufficient evidence to demonstrate a relationship between these two variables (Fig 4).

### Algorithm selection and assignment accuracy

EEG segments were DWT filtered within the delta band, since this frequency primarily contributed to ISC’s, and were assigned to 1 of the 16 clips in Experiment 1B using the aggregate correlations assignment algorithm (Fig 1c). The aggregate correlations assignment algorithm was used since it was the best performing algorithm within a separate dataset (see Experiment 1A). Using this approach, 91 out of 272 total segments (17 subjects × 16 clips), or 33.46% of the segments, were correctly assigned (chance = 06.25%). Within post-hoc analysis, we found accuracies of 08.82% and 24.26% using the separate time series and aggregate time series approach, respectively. Thus, the aggregate correlations approach was selected as the optimal approach using an independent dataset, and its utility was verified within the data in the main experiment since it continued to outperform the alternative assignment algorithms.

**Fig 5:**
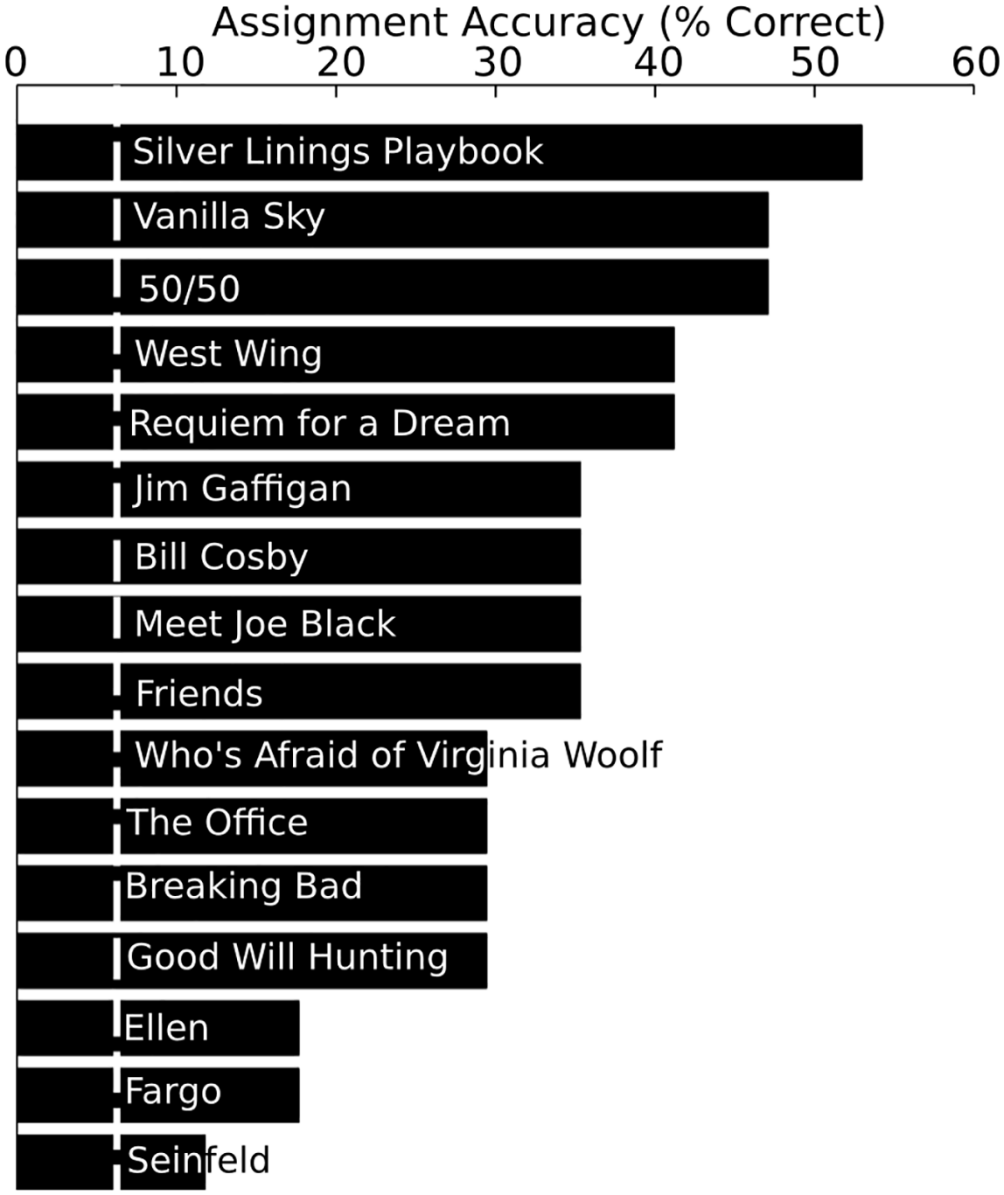
Assignment accuracy for each clip. The assignment accuracy is indicated for each clip. The clips are organized with the highest (top) to lowest assignment accuracies. The white line indicates chance assignment accuracy (06.25%).

The assignment accuracies are indicated for each of the separate clips in Fig 5. The assignment accuracies range from 52.9% (*Silver Linings Playbook*) to 11.75% (*Seinfeld*). In general, there appear to be qualitative differences in the assignment accuracies across the clips, and there is a significant relationship between delta ISC’s (in Fig 3) and assignment accuracy (in Fig 5) (r(15) = 0.76; p = 0.0007).

We examined which spatial locations were most informative for prediction by determining the percentage of correct assignments for each electrode. The best predicting electrode was electrode Cz, with 26 out of 91 (28.57%) correct predictions derived from this mid central spatial location. Electrodes Fz, O1, F3, F4, O2, P3, and P4 contributed to 10.99%, 01.10%, 10.99%, 02.20%, 17.58%, 26.37% and 02.20% of the correct predictions.

### Dependence on the number of subjects

**Fig 6:**
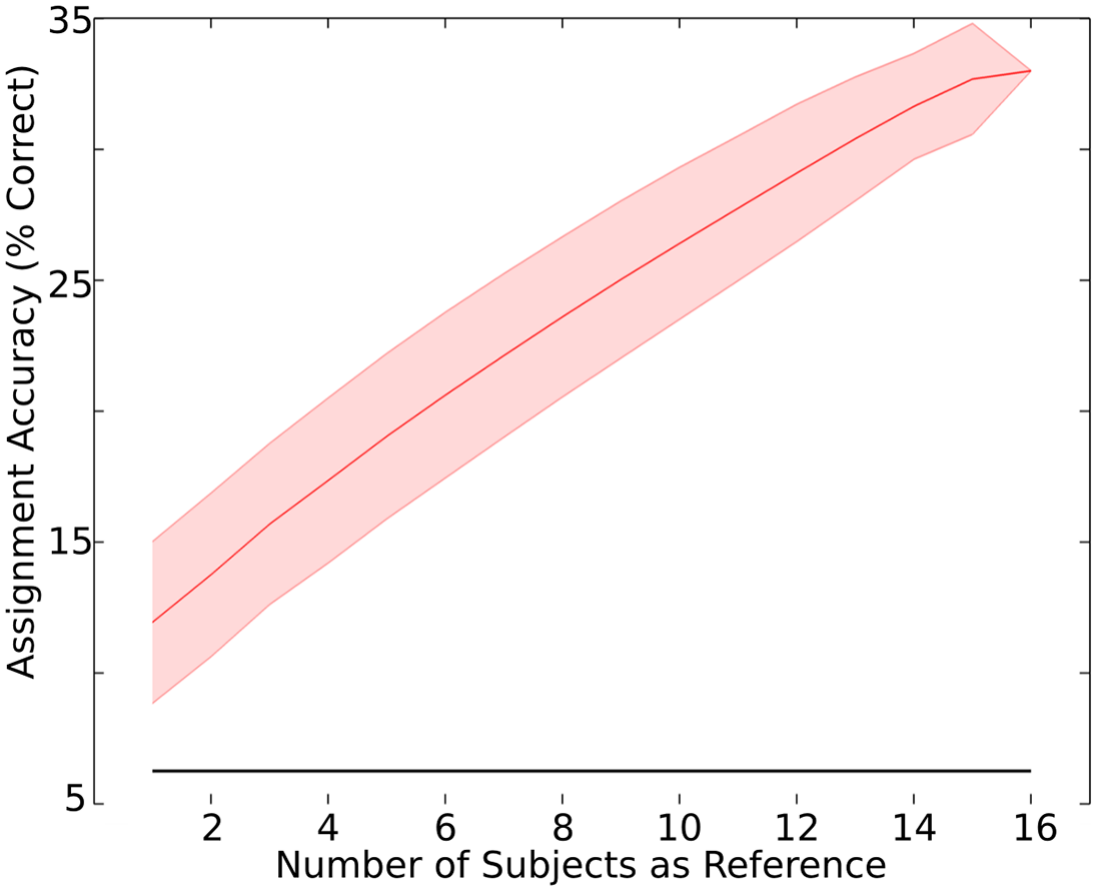
Assignment accuracy as a function of the number of subjects. The assignment accuracy was examined within the data collected in the main experiment using N = 1, 2, 3, … 16 subjects as reference. The assignment accuracy was 33.46% (chance = 06.25%, solid black line) when all 16 subjects were used as reference. The shaded area represents the standard deviation of assignment accuracies observed with repeated assignments (i.e. across the assignments generated with all unique combinations of subjects drawn from the sample of 16).

We examined assignment accuracy with the aggregate correlations approach when N = 1, 2, 3, … 16 subjects were used as reference. Assignment accuracy monotonically increased from 11.94% with N = 1, up to 33.46% with N = 16, as indicated in Fig 6. Assignment accuracy increased an average of 01.43% with each new additional subject added, and doubling the number of subjects from 8 to 16 resulted in a 41.65% increase in assignment accuracy (from 23.60% to 33.46%).

## Discussion

### Assignment accuracy for each algorithm

In the present study, our primary goal was to implement and evaluate different assignment algorithms for predicting which audiovisual clips individuals viewed. The aggregate correlations algorithm (Fig 1c) outperformed the alternative algorithms both within an independent dataset (Experiment 1A) as well as within the data obtained in the main experiment (Experiment 1B) (i.e. with a 21.4% and 37.92% increase in accuracy compared to the second best algorithm, respectively). Within the aggregate correlations algorithm, correlations were computed first for each unique subject pair, and the resulting correlations were then averaged across subjects. This approach integrates information from multiple subjects similar to the aggregate time series approach, except similarities may be preserved in the evoked potential within subject pairs that might be obscured when averaging across all subjects. For example, averaging across time series emphasizes information which is highly similar across subjects, and this constraint appears to reduce the information available when using ISC’s for prediction. The aggregate correlations approach appears most informative in the context of the present study using low magnitude EEG ISC’s, and further studies may examine whether the algorithm continues to perform better when ISC’s are obtained from signals with a higher SNR, such as with BOLD fMRI, and when compared with alternative approaches [29,30].

It is important to note that the assignment accuracy monotonically increased as the number of subjects increased. This finding suggests that further increases in assignment accuracy, from the peak at 33.46%, would likely be observed with further increases in sample size. Additional studies with a larger sample size could potentially characterize the nature of this assignment accuracy increase, revealing the subject sample that may result in a plateau in prediction accuracy. A greater sample size could additionally enhance the ability to examine whether individual differences, such as gender and the individuals prior exposure to the clip, are related to differences in ISC magnitude across clips.

### ISC’s and preference

There was insufficient evidence to support our hypothesis that individuals with similar clip preferences would have similar EEG responses (i.e. greater ISC’s). There are a few reasons why individual preferences would potentially diverge from EEG responses to the stimuli. In general, ISC magnitudes and assignment accuracies appeared to differ across the clips. These differences may be related to many aspects of the clip content, including the spectral characteristics of the auditory signals, low level characteristics of the audiovisual content and differences in attentional and emotional engagement toward the different clips. A subset of these characteristics may be related to individual’s preferences. Thus, individuals preference ratings may represent an aggregate measure of their subjective and perceptive response to the video, which may be not necessarily be related to the characteristics that lead to cortical engagement toward the stimuli, as expressed by EEG ISC magnitudes. This is consistent with previous studies demonstrating the recruitment of neural responses toward behaviorally relevant ecological events, irrespective of whether or not the content is subjectively pleasing [31]. The divergence between individual preference and cortical engagement is also consistent with a general divergence between cognitive and behavioral measures and subjective reports, as demonstrated by a recent finding that EEG ISC’s were more predictive of the preferences of a general audience than the preferences of the individuals in which the ISC’s were measured [14]. In general, this is consistent with findings which suggest that measures of neural engagement can supplement subjective reports of preference, perception, and emotion [29,32–35].

### Differences between EEG and fMRI ISC’s

The majority of ISC studies have been conducted using BOLD fMRI measures [1,6–9,12,13,29,36–50], with comparatively fewer studies using EEG or MEG [5,12,51–54]. Given this discrepancy, it is important to consider how differences in spatial and temporal sensitivities of each measure [55] may contribute to differences in the interpretation of ISC’s and the strengths and weaknesses of each modality.

Previous studies demonstrate that fMRI ISC increases correspond to segments of the clip that display content consistent with the functional properties of the region [37,41,48,56–58]. For example, early visual and auditory regions demonstrate higher ISC’s when auditory and visual content are presented, respectively [8,13,49,59], while ISC peaks within the fusiform gyrus and post-central sulcus appear to correspond to regions in the clip where faces are displayed or buildings are displayed, respectively [8]. Thus, ISC’s are a promising approach to determine complex functional properties of fMRI regions.

EEG responses arise primarily from the cortex, and the specific cortical regions of activity are difficult to localize compared to fMRI. Thus, EEG ISC’s are ill suited toward determining the functional properties of different spatial locations. Instead, EEG ISC’s emerge from a tight coupling (i.e. phase locking) of the evoked response across individuals at a given location of the clip [5]. These common modulations may emerge from phase entrainment of EEG oscillations to the sensory aspects of the clip content [60], since these events (i.e. particularly auditory speech) unfold with similar temporal properties as EEG oscillations and since they have well defined onsets and offsets [61,62]. Cognitive and emotional responses to the clip content may occur at longer time-scales than EEG oscillations and with less defined onsets and offsets, which reduces their direct contribution to EEG ISC’s. However, we anticipate that subjective engagement toward the clip contents (i.e. including cognitive and emotional responses to the clip contents) will interact with the sensory events within the clip, such that increases in engagement will be associated with increased phase locking to sensory events, and an increased ISC. Further research may disentangle these cognitive and sensory characteristics that underlie increases in ISC’s across subjects, and these findings will help determine the relationship between ISC’s and individuals perceptual, cognitive, and emotional responses to audiovisual stimuli.

### Neurologically plausible ISC magnitudes

Eye movements are coherent across subjects when viewing natural scenes [63] and eye movement artifacts strongly contribute to EEG responses measured at frontal scalp locations. Thus, it is important to consider the potential contribution of eye movement artifacts to EEG ISC’s. The contribution of these non-neurological sources may be addressed by recording eye movements directly with electrooculography (EOG), by examining the spatial locations that contribute to ISC’s, by considering the size of the video display, or by directing participants to fixate on a point at the center of the video.

EOG responses were not measured in the present study due to the limited coverage of the mobile EEG system and individual’s eye movements were not constrained to a central fixation point. However, the spatial topography of ISC’s and our choice of video size help alleviate concerns that the results of the present study are influenced primarily by eye movements. For example, the peak ISC was not observed within the electrodes which are predominantly influenced by eye movements (e.g. frontal electrodes F3, Fz, and F4 in this study). Instead, the peak ISC location (electrode Cz) is consistent with the topographic locations in which cognitive ERP peaks are often observed (e.g. the P300 [64]). Additionally, we conducted a pilot experiment to compare the magnitude of ISC’s with video and audio to the magnitude with audio only. The individual average ISC values obtained within Experiment 1A were comparable in magnitude to the individual average ISC values obtained when individuals listened to the auditory stimuli without video (S1 Fig). Thus, the use of a smaller video display size (13.2 × 7.4 dva, compared to 22 × 17 dva in [53] and 18 × 11 dva in [5] likely reduced the influence of eye movements on EEG ISC’s in the present study.

The smaller video size may have contributed to the small ISC values (i.e. r < .01) reported in the present study. These low correlations indicate that the evoked response to shared audiovisual inputs explains only a small portion of the EEG signal variance, which is expected given that the variability of single-trial evoked potentials and their low magnitude against background EEG (i.e. the poor signal to noise ratio of EEG). Previous studies have implemented spectral decomposition and canonical correlations analysis (CCA) and have reported ISC values up to 0.3. As in the present study, these studies demonstrate that ISC’s emerge from activity within the delta band (0–4 Hz), with few significant correlations observed within the higher frequencies [53,65]. Multivariate correlation methods such as CCA likely contributed to the larger ISC’s in previous studies, since the approach maximizes the correlation among paired [channels × time] matrices. Compared to the current study, the approach appears to enhance the correlations among matching as well as non-matching segments, thus, it would be interesting for future studies to examine the predictive utility of CCA using the algorithms implemented in the present study.

### The utility of EEG ISC’s for complex stimuli

The general approach for EEG analysis with complex stimuli differs considerably from conventional EEG experiments, where responses are measured to repeated presentations of a stimulus and averaged, generating an ERP. EEG responses to complex audiovisual stimuli violate many of the assumptions for traditional ERP analysis. For example, audiovisual content is typically not repeated within an individual session, which precludes multiple measurements for event-related averaging. In addition, the audiovisual content overlap in time, which may result in the superposition of phase-locked EEG oscillations arising from the responses associated with the events and the emotional and cognitive response. In contrast to ERP analysis, the ISC approach shifts emphasis from the specific shape of the EEG response toward the commonalities in the response across subjects. Thus, ERP responses that are non-stationary and non-linear with respect to a stimulus within an individual subject will contribute to large ISC values as long as the characteristics of the non-stationary and non-linear response are preserved across subjects.

It is important to note that the ISC approach may be useful for capturing complex stimulus-response characteristics that may result within conventional task-based paradigms, and thus, may complement traditional analyses of task data [9,39,39,44–46,66]. For example, there is an apparent non-linear relationship between the amplitudes of ERP responses to target tones and the number of preceding non-targets tones [67]. These amplitude fluctuations are averaged out in the conventional ERP, but would be preserved in ISC analysis, since the continuous amplitude differences would contribute to the calculation of correlation.

### Future directions

Further studies may manipulate the content of audiovisual clips and the subject population to determine whether clinical populations demonstrate similar cortical responses to particular content. For example, clinical populations may demonstrate a greater commonality in their responses among themselves than among other populations. Individuals with autism demonstrate reduced ISC’s within multiple fMRI regions while watching social interactions [47], and demonstrate reproducible, but idiosyncratic, responses amongst themselves [68]. Individuals with depression demonstrate attentional biases toward negative environmental stimuli [69,70], and these biases may emerge as enhanced ISC’s within this population (compared to controls) when viewing clips with negative content. Thus, ISC’s may be a useful approach for identifying neural response similarities that may be unique to patients, especially as we gain a better understanding of the subjective and perceptual factors that are related to ISC modulation.

## Conclusions

Within the present study, we examined the utility of using ISC’s for predicting which audiovisual clips individuals viewed, and their potential relationship to subjective reports. Three approaches were evaluated for aggregating responses across subjects for assignment within Experiment 1A. The aggregate correlations algorithm generated the highest assignment accuracy (70.83% chance = 33.33%) and was selected as the assignment algorithm for the larger sample of individuals and clips within Experiment 1B. The overall assignment accuracy was 33.46% within Experiment 1B (chance = 06.25%), with accuracies ranging from 52.9% (*Silver Linings Playbook*) to 11.75% (*Seinfeld*). ISC’s were significantly greater than zero for 15 out of 16 clips, and fluctuations within the delta frequency band (i.e. 0–4 Hz) primarily contributed to response similarities across subjects. There was insufficient evidence to indicate that individuals with greater similarities in clip preference demonstrate greater similarities in cortical responses, suggesting a lack of association between ISC and clip preference. These results demonstrate the utility of using ISC’s for prediction, and further characterize the relationship between ISC magnitudes and subjective reports.

## Supporting Information

S1 Fig Average ISC’s with and without video. ISC’s in Experiment 1A were computed for each clip and subject pair for the three frontal electrodes (F3, Fz, and F4). Individual average ISC’s were computed by averaging the ISC’s computed for the 3 clips and 3 electrodes for each unique session pair. The column “Video + Audio” indicates the distribution of individual ISC’s in Experiment 1A with video and audio. The column “Audio” indicates the distribution of individual ISC’s when the experiment was repeated with only audio (i.e. without video, and while individuals fixated). There was insufficient evidence of a different in ISC’s between the two conditions (T(10) = -0.09; p = 0.93). These results suggest that eye movements were not a strong contributor to ISC’s within the present study.
(PNG)

S2 Fig Overall average ISC for each electrode. The spatial distribution of ISC’s were examined by computing the average ICS (across all subject pairs and clips) separately for each electrode. The 8 electrodes are indicated by their 10–20 label (i.e. F3, Fz, F4, Cz, P3, P4, O1, and O2) with the average ISC indicated below each label. The highest ICS’s were observed over the mid central electrode (Cz) and the electrode located over occipital regions (O2).
(PNG)
