# Supplementary figures and images for: Cortical Response Similarities Predict which Audiovisual Clips Individuals Viewed, but Are Unrelated to Clip Preference
Source: PLoS One. 2015 Jun 1;10(6):e0128833. doi: 10.1371/journal.pone.0128833 (PMC4452623; doi:10.1371/journal.pone.0128833)

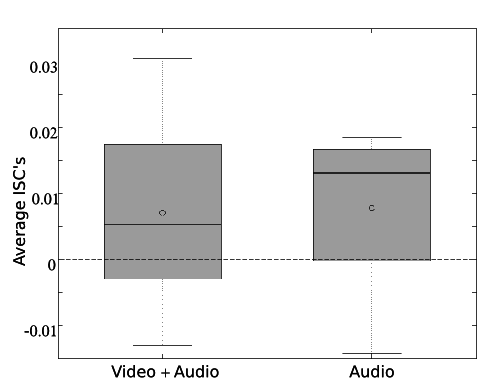

Supplement: S1 Fig — ISC’s in Experiment 1A were computed for each clip and subject pair for the three frontal electrodes (F3, Fz, and F4). Individual average ISC’s were computed by averaging the ISC’s computed for the 3 clips and 3 electrodes for each unique session pair. The column “Video + Audio” indicates the distribution of individual ISC’s in Experiment 1A with video and audio. The column “Audio” indicates the distribution of individual ISC’s when the experiment was repeated with only audio (i.e. without video, and while individuals fixated). There was insufficient evidence of a different in ISC’s between the two conditions (T(10) = -0.09; p = 0.93). These results suggest that eye movements were not a strong contributor to ISC’s within the present study. (PNG) [file pone.0128833.s001.png]

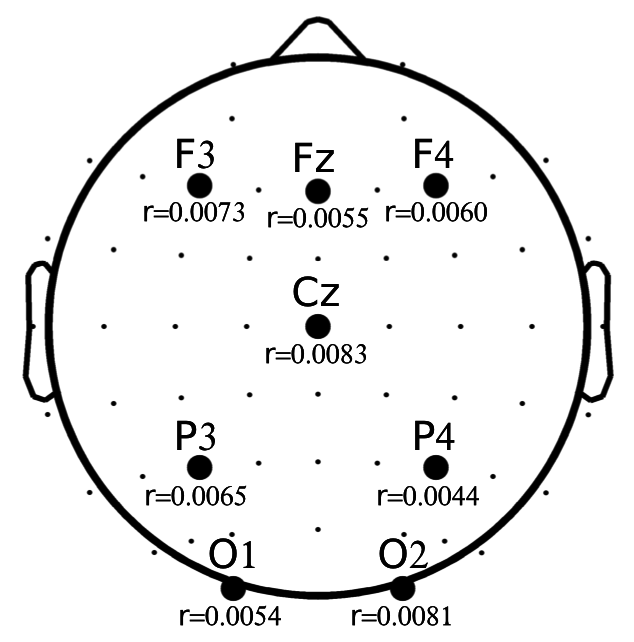

Supplement: S2 Fig — The spatial distribution of ISC’s were examined by computing the average ICS (across all subject pairs and clips) separately for each electrode. The 8 electrodes are indicated by their 10–20 label (i.e. F3, Fz, F4, Cz, P3, P4, O1, and O2) with the average ISC indicated below each label. The highest ICS’s were observed over the mid central electrode (Cz) and the electrode located over occipital regions (O2). (PNG) [file pone.0128833.s002.png]
